# From Phage Display to Yeast Secretion: Developing Fc-Fused Nanobodies Against Influenza Virus

**DOI:** 10.3390/cells15080655

**Published:** 2026-04-08

**Authors:** Mei Wang, Shujun Li, Yong Li, Xiaomei Xia, Yan Zhang, Ning Cao, Yuanfang Li, Yijia Liu, Sheng Zhang, Lilin Zhang, Jinhai Huang

**Affiliations:** 1 School of Life Sciences, Tianjin University, Tianjin 300072, China; 2023226011@tju.edu.cn (M.W.); 15863090326@163.com (S.L.); yong.li@tju.edu.cn (Y.L.); xiaomeixia612@126.com (X.X.); jennerzy@163.com (Y.Z.); caoningzai@126.com (N.C.); 2020226010@tju.edu.cn (Y.L.); jiaawd@163.com (Y.L.); 2 Tianjin Zoo, Tianjin 300070, China; 15522272608@163.com

**Keywords:** influenza, VHH, IgY Fc fusion, nanobody, phage display, hemagglutinin, neuraminidase, *Saccharomyces cerevisiae*, VHH-Fc fusion

## Abstract

**Highlights:**

**What are the key innovations and advantages of this study?**

- Phage display using H9N2-infected MDCK cells enables isolation of VHHs recognizing native HA and NA, preserving conformational epitopes for accurate functional selection.
- Yeast-expressed VHH-Fc fusions were efficiently secreted at 15–20 mg/L, demonstrating the system’s high-yield production capability.
- This study presents a novel modular format for antiviral antibody development against AIV by fusing camelid VHHs with chicken IgY Fc for the first time.

**What are the main findings?**

- VHHs isolated from H9N2-infected cells recognize native HA and NA.
- Selected VHHs target conserved HA stalk/stem and NA active sites.
- Docking predicts key CDR and framework residues contributing binding.
- Yeast-expressed VHH-Fc fusions secreted efficiently at 15–20 mg/L.
- VHH-Fc clones neutralize virus, inhibit NA, and bind multiple HA subtypes.

**What are the implications of the main findings?**

- Native-targeting VHHs increase likelihood of functional neutralization in vivo.
- Binding conserved HA/NA regions supports potential cross-subtype antiviral activity.
- Framework residues contribute to structural recognition across multiple influenza subtypes.
- Yeast expression enables high-yield, scalable production of functional VHH-Fc antibodies.

**Abstract:**

Avian influenza infections cause substantial economic losses in the poultry industry and raise public health concerns due to viral adaptation and cross-species transmission. The frequent antigenic drift of influenza viruses further complicates the prevention and treatment of avian respiratory infections. In this study, we generated high-affinity heavy-chain variable domain (VHH) nanobodies from naïve alpaca/camelid VHH libraries using phage display combined with H9N2 *influenza A virus* (IAV)-infected Madin-Darby Canine Kidney (MDCK) cells. Based on binding affinity and neutralization potential, we identified seven hemagglutinin (HA)-specific and two neuraminidase (NA)-specific VHHs. Molecular docking predicted the interaction sites of HA-specific VHHs (L1-2, L1-4, A5) and NA-specific VHHs (L1-3, L2-2), providing mechanistic insights. Notably, the three HA-specific VHHs (L1-2, L1-4, A5) showed cross-reactivity to representative HA subtypes (H1, H3, and influenza B), indicating recognition of conserved epitopes across divergent influenza strains. For the first time, these camelid nanobodies were fused to the chicken IgY Fc domain, and the expression cassette was integrated into the *Saccharomyces cerevisiae* genome, achieving a secretion yield of 15–20 mg/L of VHH-Fc antibodies. Experimental validation confirmed that the three HA-specific VHHs-Fc constructs effectively blocked viral infection, while the two NA-specific VHH-Fc constructs (L1-3, L2-2) inhibited NA activity, demonstrating the functional efficacy of the yeast-secreted VHH–IgY Fc platform. This novel IgY Fc fusion approach offers a scalable platform with enhanced stability, extended circulation potential, and applicability in poultry.

## 1. Introduction

Influenza remains a persistent global health challenge due to high viral mutation and recombination rates, which severely limit the development of universal vaccines and commercial antiviral therapies [1]. As a member of the *Orthomyxoviridae* family, *influenza A virus* (IAV) is a segmented, enveloped, negative-sense RNA virus with 18 hemagglutinin (HA) and 11 neuraminidase (NA) subtypes, among which H5, H7, and H9 subtypes are the most pathogenic to avian populations [2,3]. In particular, the avian-origin H9N2 subtype has emerged as a critical zoonotic pathogen with dual implications for public health and the poultry industry [4,5]. Annually, H9N2 causes over $1.2 billion in global economic losses due to elevated avian mortality, international trade bans, and high vaccine procurement costs [1]. More critically, it weakens the avian immune system, increasing susceptibility to secondary infections and exacerbating poultry industry crises [6]. Despite widespread use of inactivated vaccines, H9N2 IAVs remain dominant in chicken flocks. Vaccines fail to block viral replication in the upper airway and cannot keep pace with the virus’s high mutation rate (≈1.2 × 10^−3^ substitutions/site/year) that drives immune evasion and enhanced viral replication [4,5,7,8]. Furthermore, H9N2 can undergo genetic reassortment with other IAV subtypes to generate novel pandemic strains (e.g., H7N9) [9], underscoring the urgent need for innovative antiviral strategies.

The viral surface glycoproteins HA and NA are core targets for antiviral interventions. HA mediates viral attachment and host cell entry via membrane fusion [10], while NA facilitates virion release and dissemination by cleaving sialic acid residues [11]. Neutralizing antibodies targeting HA and NA can inhibit IAV infection [12,13]. Higher systemic titers of HA- and NA-binding antibodies correlate with protection against clinical influenza [13], and antibodies targeting conserved epitopes of these glycoproteins can confer broad-spectrum cross-protection. Traditional chicken egg yolk antibodies (IgY) have been explored for anti-influenza applications given their non-mammalian origin and low cross-reactivity, but they face inherent limitations, including potential exogenous viral contamination (e.g., avian influenza virus), highly non-specific impurities (e.g., lipid residues), product heterogeneity from polyclonal mixing, and handling stress to waterfowl such as ducks and geese [14,15], necessitating the development of alternative antibody-based tools.

In this context, camelid-derived variable domains of heavy-chain-only antibodies (VHHs, also called nanobodies or single-domain antibodies, sdAbs) have garnered widespread attention in antiviral drug development in recent years [3,16,17]. Distinguished from conventional antibodies by their small molecular weight (15 kDa), high stability, strong tissue penetration, and unique extended complementarity-determining region 3 (CDR3) that enables recognition of cryptic epitopes inaccessible to traditional antibodies, VHHs exhibit unique advantages in targeting viral surface antigens [17,18]. Moreover, VHHs possess excellent genetic manipulability, enabling the construction of fusion proteins with immunoglobulin Fc fragments that combine VHH-specific antigen binding with Fc-mediated effector functions (e.g., complement-dependent cytotoxicity, antibody-dependent cellular cytotoxicity), while also extending the circulating half-life of the fusion protein independent of these effector functions [18,19]. Previous studies have demonstrated that VHH-Fc fusion antibodies can enhance pharmacokinetic properties and therapeutic efficacy, making them promising candidates for combating emerging and re-emerging viral infections [17,20]. Notably, chicken IgY Fc fragments, with their unique structural and functional characteristics, offer an alternative scaffold for VHH fusion, potentially expanding the applicability of sdAb-based therapeutics in both veterinary and human medicine [21,22].

Phage display technology has become a mature and efficient platform for screening target-specific VHHs from naïve or immune libraries [23,24], enabling rapid enrichment of antigen-binding clones through multiple rounds of panning followed by validation of binding specificity and functional activity [25]. However, screening VHHs against HA and NA remains challenging because these glycoproteins form oligomers in their native viral state (HA as trimers, NA as tetramers), and recombinant soluble antigens may not fully preserve conformation-dependent epitopes. While naïve libraries can yield functional VHHs without immunization, immunization followed by library construction has also successfully produced potent VHHs recognizing native HA and NA [26,27]. Thus, both immunization-dependent and -independent approaches are feasible, but capturing epitopes in their native oligomeric conformation remains a key technical challenge. For heterologous expression of recombinant VHH fusion antibodies, *S. cerevisiae* has emerged as a safe, cost-effective, and scalable expression host, capable of secreting correctly folded and post-translationally modified proteins (e.g., glycosylation); the use of cell wall-deficient yeast strains further enhances extracellular protein secretion, facilitating large-scale production of functional VHH fusion antibodies [28,29,30].

Although camel VHH-based antiviral agents have attracted increasing interest, studies systematically generating VHH–IgY Fc fusions against influenza HA or NA while preserving native conformational epitopes remain limited, and the relationship between antigen binding specificity and functional inhibition has not been fully established [31]. To overcome the challenge of preserving conformational epitopes, we constructed a naïve camelid VHH phage display library and applied a combined positive selection and negative subtractive panning strategy using H9N2-infected MDCK cells as the antigen source. This approach maintained native trimeric HA and tetrameric NA structures during selection [32].

Screening yielded seven HA-specific VHHs (targeting H9-HA) and two NA-specific VHHs (targeting N2-NA), selected based on binding affinity and neutralizing potential. Five lead candidates were reformatted as VHH-IgY Fc fusion proteins and expressed in the cell wall-deficient yeast strain *S. cerevisiae* SJC1. Fusion to the IgY Fc domain was designed to: (1) enhance avidity and extend serum half-life via dimerization, addressing the short circulatory persistence of monomeric VHHs; and (2) enable proper post-translational modification in a cost-effective yeast secretion system while preserving bioactivity. Recombinant VHH-IgY Fc antibodies were evaluated for binding specificity and cross-reactivity by indirect ELISA and for NA inhibitory activity using a fluorescence-based MUNANA assay. The fusion antibodies exhibited moderate virus-neutralizing activity against H9N2. Notably, the IgY Fc domain improved stability in mucosal environments (e.g., the gastrointestinal tract), supporting potential mucosal delivery applications [33].

This study aimed to generate VHH-IgY Fc chimeric antibodies with specific binding to H9N2-HA/NA and potent NA inhibition, providing both theoretical insight and experimental evidence for novel anti-H9N2 biologics. Our findings lay a foundation for antibody-based strategies to combat *influenza A viruses*, with implications for future diagnostic and therapeutic development.

## 2. Materials and Methods

### 2.1. Cell Lines and Viruses

MDCK cells were cultured in DMEM supplemented with 10% fetal bovine serum and 1% penicillin/streptomycin. Cells were maintained at 37 °C in a 5% CO_2_ incubator. The H9N2 influenza virus strain (Hebei/L1/2006) was propagated in MDCK cells, and viral titers were determined by the 50% tissue culture infectious dose (TCID_50_) assay using the Reed–Muench method [34]. Serum neutralization 50% protective dose (PD_50_) values were determined using serial dilution of yeast culture supernatant containing secreted VHH-Fc fusions. All procedures were conducted in BSL-2 conditions.

### 2.2. Construction of Naïve VHH Phage Display Libraries

Peripheral blood mononuclear cells (PBMCs) were isolated from 10 mL heparinized blood of three non-immunized camelids (one alpaca, two llamas) housed at Tianjin Zoo (Tianjin, China). Total RNA was extracted from PBMCs, and complementary DNA (cDNA) was synthesized using the HiScript III 1st Strand cDNA Synthesis Kit (Vazyme, Nanjing, China) as the template for nested PCR amplification of VHH genes, as described previously [35]. For nested PCR: the first round used primers CALL-F and CALL-R to amplify the ~750 bp heavy-chain antibody (HCAb) variable region precursor. The second round employed primers VHH-SfiI-F and VHH-NotI-R (shown in Table 1) for specific amplification of the ~500 bp VHH fragment. PCR products were separated by 1% agarose gel electrophoresis, and the 500 bp VHH band was purified using a Gel Extraction Kit (Omega Bio-tek, Norcross, GA, USA). The purified VHH fragment was cloned into the SfiI/NotI-pre-digested pCANTAB 5E vector via seamless cloning. Recombinant plasmids were transformed into *Escherichia coli* TG1 competent cells by electrotransformation. The transformed cells were subsequently recovered in Luria–Bertani (LB) medium at 37 °C with shaking at 180 rpm for 1 h. To determine library size, 100 μL aliquots of transformed cells were serially diluted (10^−4^–10^−6^), spread onto LB agar plates supplemented with ampicillin and 2% glucose, and cultured overnight at 37 °C. Colony-forming units (CFUs) were counted, and library capacity was calculated as: Library size (CFU) = (Average CFU × Dilution factor × Total volume of transformed culture)/Volume plated. Glycerol stocks (50% *v*/*v*) were prepared and stored at −80 °C for long-term preservation.

Recombinant phages were rescued by infecting library-containing TG1 cells with an MOI of 20 (multiplicity of infection) of M13KO7 helper phages (New England Biolabs, Ipswich, MA, USA) at 37 °C for 40 min. Infected cells were cultured overnight in LB medium with ampicillin and kanamycin, and phages were concentrated from the supernatant by polyethylene glycol (PEG)/NaCl precipitation [36]. This procedure was performed independently for each camelid, yielding three separate VHH phage display libraries.

### 2.3. Specific H9N2 VHH Antibodies Screening by Virus-Infected MDCK Cells

Following construction of the naïve VHH library, phage display selection was performed on MDCK cells expressing H9N2-HA/NA using a binding-dissociation strategy of positive selection and negative subtractive panning as the following steps. For positive panning, MDCK cell monolayers infected with influenza A H9N2 virus were used as targets—viral infection induces surface expression of native HA and other envelope proteins with conformationally native epitopes accessible to phage-displayed VHHs [32]. The phage library was added at 1 × 10^12^ pfu/mL, followed by incubation at 4 °C for 45 min. Cell monolayers were washed five times with PBS, and bound phages were eluted with buffer (pH 4.5, 500 mM NaCl). Eluted phages were neutralized and subjected to negative selection by incubation with uninfected MDCK cells under the same conditions (4 °C, 45 min). Three rounds of panning were performed. Subsequently, 30 individual clones were randomly selected for PCR validation and Sanger sequencing (Genewiz, Suzhou, China). Complementarity-determining regions (CDRs) were identified using MEGA 11.0 software.

Phage enzyme-linked immunosorbent assay (ELISA) was conducted to screen positive binders. Recombinant H9-HA protein (H9N2 strain, Shijiazhuang, China) was used as the coating antigen, with bovine serum albumin (BSA; Sigma-Aldrich, St. Louis, MO, USA) and M13KO7 helper phage as negative controls. Horseradish peroxidase (HRP)-conjugated anti-M13 IgG (Beijing Yiqiao Shenzhou, Beijing, China) was employed for detection. Clones with an optical density at 450 nm (OD_450_) ≥0.5 and a positive-to-negative (P/N) ratio >2 were defined as positive binders.

### 2.4. Construction of Recombinant S. cerevisiae Strains Expressing VHH-Fc Antibodies

The chicken IgY heavy-chain sequence (GI: 2315625892) was retrieved from the NCBI database. Using chicken cDNA as the template, CH2-CH3-CH4 Fc fragments were amplified with primers IgY Fc-F (5′-GGCTGAAGCTCCCGGGTCTAGACA CCCCTCCTCCTGCACCCCG-3′) and IgY Fc-R (5′-TCAGTGGTGGTGGTGG TGGTGTTTACCAGCCTGTTTCTG-3′). The amplicon was used to construct the POT-IgY Fc vector, a dedicated plasmid for IgY Fc fusion protein expression.

Selected VHHs were amplified with seamless cloning primers α-VHH-F (5′-GAGGCTGAAGCTCCCGGGCAGGTGCAGCTGGTAGAG-3′) and IgY-VHH-R (5′-GGTGCAGGAGGAGGGGTGTGAGGAGACGGTGACCTG-3′), which introduce homologous arms complementary to the POT-IgY Fc vector. VHH amplicons were inserted into the SmaI-linearized POT-IgY Fc vector via seamless cloning using the ClonExpress II One-Step Cloning Kit (Vazyme, Nanjing, China) [37]. Recombinant constructs contained an N-terminal α-factor secretion signal (for extracellular secretion) and a C-terminal 6 × His tag (for downstream detection and purification).

To construct VHH-Fc fusion protein expression cassettes: recombinant plasmids were digested with BsaI to excise transcriptional units (TUs) encompassing the full VHH-Fc expression cassette; separately, plasmids containing homology arms and a tryptophan (*Trp*) selection marker were digested with BsmBI to generate fragments of the left homology arm, *Trp* marker, and right homology arm. These fragments were ligated overnight at 16 °C with T4 DNA ligase to assemble full integration cassettes (configuration: left homology arm–VHH-Fc–Trp–right homology arm).

For efficient secretory expression, the cell wall-deficient yeast strain SJC1 (genotype: MATa *his3Δ200 leu2Δ0 lys2Δ0 trp1Δ63 ura3Δ0 met15Δ0 chs3Δ*) was selected as the host—deletion of *chs3* (encoding chitin synthase III) compromises cell wall integrity, facilitating enhanced extracellular secretion of recombinant proteins [38]. Lithium acetate (LiAc)-mediated transformation was employed for targeted integration of VHH-Fc cassettes into three distinct loci on chromosomes IV and XVI of SJC1, generating recombinant strains harboring three copies of the VHH-Fc expression cassette to ensure stable and elevated fusion protein production.

### 2.5. Expression, Concentration and Quantification of VHH-Fc Fusion Proteins

Recombinant yeast clones were cultured in 100 mL YPD medium at 30 °C with agitation (200 rpm) for 72 h. The culture supernatant was collected by centrifugation (4 °C, 8000 rpm for 30 min) to remove yeast cells. For preliminary purification and concentration, the supernatant was loaded onto 10 kDa molecular weight cutoff ultrafiltration tubes (Thermo Fisher Scientific) and centrifuged (4 °C, 3900 rpm for 20 min) to concentrate the sample 10-fold. During this process, the buffer was repeatedly exchanged with 1 × PBS to remove residual medium components (e.g., yeast metabolites).

The concentrated supernatant was subsequently applied to a Ni^2+^-NTA affinity column (Thermo Fisher Scientific, Waltham, MA, USA) to purify His-tagged VHH-Fc fusion proteins. Bound proteins were washed with PBS containing 20 mM imidazole and eluted with 250 mM imidazole in PBS. Purified samples were filtered through 0.22 μm sterile filters and stored at −80 °C. The relative purity and approximate concentration of VHH-Fc fusion proteins were evaluated by 12% SDS-PAGE (Coomassie brilliant blue staining), and the exact protein concentration was determined using a BCA Protein Assay Kit (Beyotime, Shanghai, China).

### 2.6. Western Blotting

Concentrated supernatants were mixed with 5× loading buffer, boiled at 100 °C for 10 min, and resolved by 12% SDS-PAGE. Proteins were transferred onto methanol-activated PVDF membranes (PALL, Port Washington, NY, USA) via wet transfer (300 mA, 100 min). Membranes were blocked with 5% skim milk in PBST (1× PBS, 0.05% Tween-20) for 1 h at room temperature, followed by incubation with rabbit anti-His-tag antibody (1:5000; Yeasen Biotech, Shanghai, China) at 4 °C overnight. After three washes with PBST (10 min each), membranes were incubated with goat anti-rabbit IgG-HRP (1:5000; Yeasen Biotech, Shanghai, China) for 1 h at room temperature. Signal detection was performed using a ChemiDoc Imaging System (Bio-Rad, Hercules, CA, USA). This assay verified VHH-Fc expression via the His-tag.

### 2.7. Modeling Antibody Crosslinking from Structural Information

Molecular docking simulations were conducted using atomic coordinates of HA and NA fragments from various subtypes obtained from the Protein Data Bank (PDB). Structural homology models were generated with Swiss-Model (https://swissmodel.expasy.org/, accessed on 23 July 2025) and SAVE v6.0 (https://saves.mbi.ucla.edu/, accessed on 23 July 2025). Active residues involved in antibody interactions were identified via CPORT (https://github.com/haddocking/cport, accessed on 24 July 2025). Docking between HA/NA and antibodies was performed using HADDOCK 2.4 (http://bianca.science.uu.nl/haddock2.4/, accessed on 25 July 2025).

Dissociation constants (Kd, M) and binding free energies (ΔG, kcal/mol) for antibody-HA/NA complexes were predicted using PRODIGY (http://wenmr.science.uu.nl/prodigy/, accessed on 26 July 2025), which estimates binding affinity from docking models. These predicted Kd values served as references for selecting candidate antibodies for further functional validation.

### 2.8. ELISA

Plates were coated overnight at 4 °C with 5 μg/mL of recombinant HA proteins: H9-HA (H9N2, Shijiazhuang, China; signal peptide and transmembrane domain removed, recombinant expression), H1-HA (A/Victoria/2570/2019, H1N1), H3-HA (A/Darwin/9/2021, H3N2), and B-HA (B/Austria/1359417/2021, Victoria lineage). After PBST washes (3×, 5 min each), plates were blocked with 3% BSA at 37 °C for 1 h. Phage samples or 10-fold concentrated yeast supernatants (VHH-Fc) were added and incubated at 37 °C for 30 min. Following PBST washing (3×, 5 min each), Bound VHHs were detected using HRP-conjugated antibodies: mouse anti-M13 (monoclonal; Beijing Yiqiao Shenzhou, Beijing, China) for phage ELISA, and goat anti-chicken IgG-HRP (1:5000; Beijing Boaosen Biotechnology Co., Ltd. Beijing, China) for yeast-secreted VHH-Fc. Color development was performed with TMB substrate, terminated with 2 M H_2_SO_4_, and absorbance was measured at 450 nm. Negative controls included an irrelevant VHH-Fc fusion protein (to exclude non-specific Fc or scaffold binding) and M13KO7 helper phage (as phage background reference). Background was confirmed to originate from residual non-specific protein interactions rather than antigen stickiness. P/N value is calculated as the ratio of the absorbance (OD_450_ nm) of the experimental group (P) to the absorbance of the negative control group (N). The negative control group for phage ELISA is M13KO7 helper phage, and for fusion protein ELISA is irrelevant VHH-IgY Fc fusion antibody. A P/N value ≥ 2.0 is defined as positive binding, indicating specific interaction between the antibody/phage and the target antigen.

### 2.9. NA Activity Inhibitor Assay

NA activity inhibition was evaluated using a fluorescence-based neuraminidase inhibition (MUNANA) assay [39], with purified N2-NA antigen (from H9N2/Shijiazhuang China strain, isolated in our lab) and VHH-Fc fusion antibodies. VHH-Fc antibodies were serially diluted 2-fold in 1× PBS (pH 7.4). Reactions were established in 96-well black microplates by mixing 50 μL of diluted antibody with 50 μL of N2-NA (final concentration 0.5 μg/mL), and incubating at 37 °C for 30 min. Controls included: (1) heat-inactivated VHH-Fc (95 °C for 10 min), (2) 1× PBS (maximum NA activity); (3) irrelevant VHH-Fc (non-NA-specific) to exclude non-specific inhibition. After incubation, 100 μL of NA fluorescent substrate (MUNANA; Beyotime, Shanghai, China, 25 μM) was added to each well, and the mixture was incubated at 37 °C for 1 h in the dark. The reaction was terminated by adding 100 μL of stop buffer (0.1 M glycine-NaOH, pH 10.7). Fluorescence intensity was measured using a microplate reader. Relative NA activity was calculated as (fluorescence intensity of test group/PBS control) × 100%, and NA inhibition rate (%) as 100% minus relative NA activity. The 50% inhibitory concentration (IC_50_) of VHH-Fc antibodies was determined by nonlinear regression to quantify NA inhibitory activity.

### 2.10. Viral Neutralization Assay

MDCK cells were seeded in 96-well plates and cultured until reaching 90% confluency. The H9N2 virus was serially diluted 10-fold in DMEM supplemented with 1% fetal bovine serum, and 100 μL of each dilution was added to eight replicate wells. Plates were incubated at 37 °C with 5% CO_2_ for 48 h, after which cytopathic effects (CPE) were observed. TCID_50_ values were calculated using the Reed–Muench method.

VHH-Fc antibodies (10-fold concentrated as described in Section 2.5) were serially diluted 2-fold in maintenance medium. Fifty microliters of diluted antibody was mixed with 50 μL of *H9N2* virus at a final concentration of 200 TCID_50_ and incubated at 37 °C for 1 h. Confluent MDCK monolayers were washed twice with PBS before adding 100 μL of the antibody–virus mixture per well (eight replicates). After 1 h of adsorption at 37 °C with 5% CO_2_, 100 μL of maintenance medium was added, and plates were incubated for an additional 96 h. Wells without CPE were considered protected. The 50% protective dose (PD_50_) was calculated using the Reed–Muench method [40]. To control for variability in yeast expression, all VHH-Fc samples were quantified by BCA to ensure consistent protein input.

### 2.11. Statistics, Replicates, and Software

All experiments were conducted with a minimum of two biological replicates, and representative data are presented herein. Statistical analyses were performed using GraphPad Prism 9. Biological replicates comprised independently infected and treated cells. Data are expressed as mean ± standard deviation (SD), with statistical significance set at *p* < 0.05.

## 3. Results

### 3.1. Construction of Three Camelid Naïve VHH Libraries

**Figure 1:**
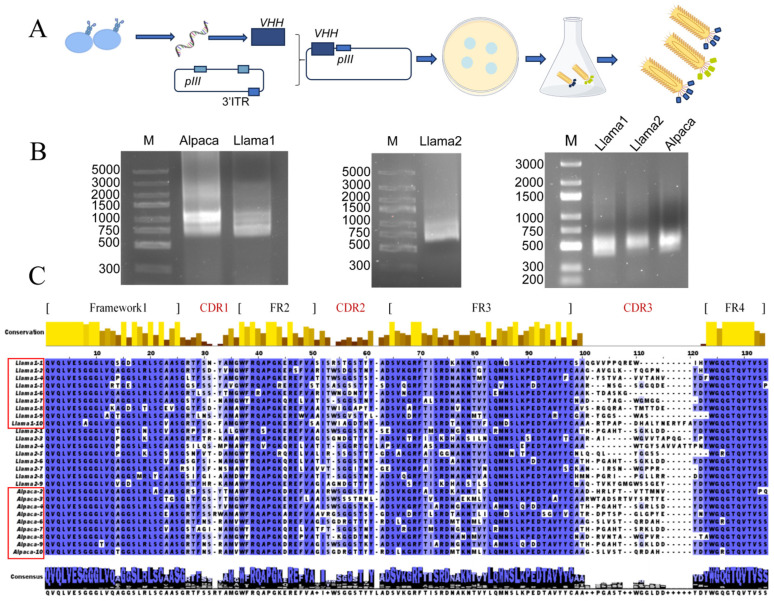
Construction of three naïve VHH libraries. (**A**) Library Generation Workflow: RNA extracted from PBMCs of Vicugna pacos and Lama glama was reverse-transcribed into complementary DNA, followed by nested PCR of the VHH domains. The resulting amplicons were cloned into the pCANTAB 5E vector (VHH gene inserted into MCS upstream of pIII, expressing VHH as a pIII N-terminal fusion) and subsequently transformed into TG1 *E. coli* cells to construct a phage-display library. Here, VHH acts as the surface-displayed target (retaining antigen-binding activity for screening), while pIII anchors VHH to phages, couples VHH phenotype to its genotype, and maintains phage infectivity for library amplification. (**B**) Agarose Gel Electrophoresis of Nested PCR Products: The initial PCR yielded 750 bp heavy-chain antibody fragments, while the subsequent PCR produced 450 bp VHH amplicons from both Llama and Alpaca samples. (**C**) CDR3 Sequence Diversity Analysis: Amino acid alignments demonstrated extensive variability in complementarity-determining regions across the three libraries. Blue indicates high conservation. The bar chart above illustrates residue conservation, with yellow indicating high conservation and brown indicating low conservation.

Three naïve VHH phage display libraries were constructed from PBMCs of two llamas (L1, L2) and one alpaca (A) (Figure 1A). Nested PCR amplified VHH fragments (450 bp) (Figure 1B), which were cloned into the pCANTAB 5E vector and transformed into *E. coli* TG1 cells. Library size assessment revealed that the llama L1, L2, and alpaca A VHH libraries comprised approximately 6.3 × 10^8^ CFU, 2.39 × 10^9^, and 2 × 10^9^ colony-forming units (CFU), respectively. Colony PCR analysis confirmed that the VHH gene insertion positive rate ranged from 90% to 100% across the libraries (Figure S1), indicating that both library capacity and insertion efficiency meet the criteria necessary for effective downstream screening of antigen-specific VHHs.

Sequencing and subsequent analysis of randomly selected clones revealed that each library contained nine distinct VHH sequences (Figure 1C). The complementarity-determining region 3 (CDR3) exhibited considerable variability, with amino acid lengths spanning 10 to 26 residues and displayed diverse amino acid compositions. The framework regions (FRs) preserved the canonical camelid VHH features [41] while also displaying the GLEW motif typically found in conventional VH frameworks (Figure S2). Collectively, these findings indicate that the constructed naïve VHH library maintains both substantial genetic diversity and sequence integrity, providing a robust foundation for the downstream isolation of VHHs targeting the native H9N2 influenza virus antigen.

### 3.2. Characterization of VHH-Displaying Phages Against Influenza Virus

**Figure 2:**
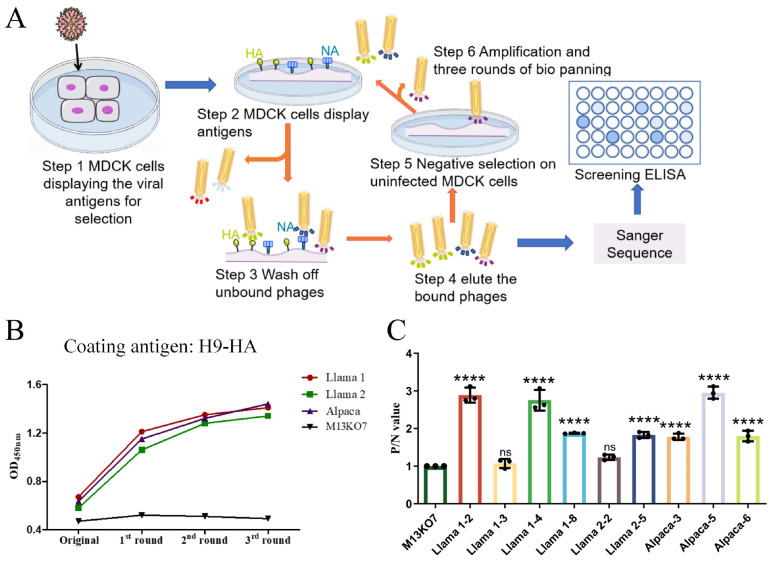
Enrichment and specificity of VHH-displaying phages. (**A**) Schematic representation of three rounds of phage panning on H9N2-infected MDCK cells with negative selection on uninfected cells. (**B**) Binding curves of phages from each panning round measured by ELISA against H9-HA. X-axis: Panning rounds; Y-axis: OD_450_, Phage titer used for ELISA was 5 × 10^11^ pfu/mL in all groups. Progressive enrichment of HA-binding phages is observed across rounds; M13KO7 control remained at baseline. (**C**) ELISA reactivity: Bar graphs illustrating the specificity profiling of clones against H9-HA and N2-NA revealed that seven clones exhibited strong reactivity to H9-HA, whereas two clones (L1-3 and L2-2) displayed specificity for NA (P/N > 2.0). **** indicates *p* < 0.0001, and “ns” indicates no significant difference (*p* ≥ 0.05) compared with the negative control (M13KO7).

To isolate VHH antibodies capable of recognizing the native conformational antigen of H9N2 virus, H9N2-infected MDCK cells were employed as the positive selection target, while uninfected MDCK cells served as the negative control during three rounds of biopanning across three naïve VHH libraries (Figure 2A, Table S1). Phage ELISA analysis demonstrated a progressive increase in HA-binding signals with successive selection rounds, whereas the M13KO7 helper phage consistently remained at baseline, confirming the effective enrichment of HA-reactive phage clones (Figure 2B).

From the outputs of these three selection rounds, 22 PCR-positive clones were sequenced, yielding 9 unique VHH sequences (Figure S3). Subsequent specificity assessment via phage ELISA revealed that seven sequences exhibited strong binding to H9-HA (OD_450_ ≥ 0.5, P/N > 2), whereas two sequences (L1-3, L2-2) showed weak H9-HA signals, indicative of N2-NA specificity (Figure 2C). Among the HA-specific clones, L1-2, L1-4, and A5 displayed the highest binding intensities, suggesting they are high-affinity candidates. Based on binding strength, sequence uniqueness, and target specificity, five core candidate clones were prioritized for downstream analyses (HA-specific: L1-2, L1-4, A5; NA-specific: L1-3, L2-2). These clones will undergo subsequent structural modeling, fusion expression, and functional validation to ensure both representativeness and antigen specificity in the study.

### 3.3. Predicted VHH–Antigen Interactions

**Figure 3:**
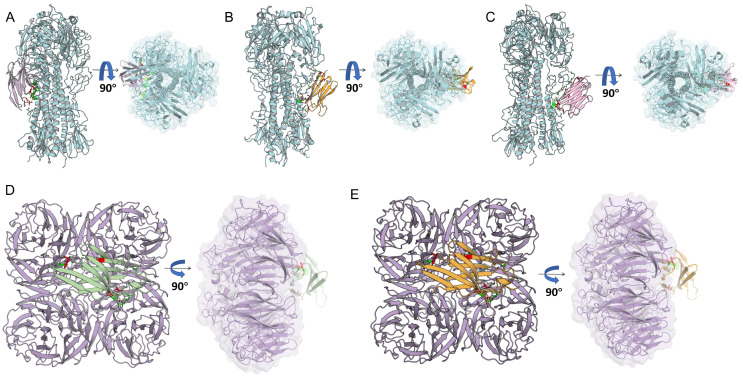
Predicted interactions of VHHs with H9N2 HA and NA. (**A**–**C**) Predicted binding of HA-targeting VHHs (L1-2, L1-4, A-5) to HA stem region from molecular docking simulations. Docking provides hypothetical binding modes for reference. (**D**,**E**) Predicted binding of NA-targeting VHHs (L1-3, L2-2) to NA surface loops adjacent to the enzymatic site. Binding interfaces are for comparative purposes only. Green stick residues represent amino acids on the antigen involved in hydrogen bond formation; red stick residues represent hydrogen bond-forming amino acids on the VHH paratope; and yellow dashed lines indicate the predicted hydrogen bonds between the VHH and antigen.

To elucidate the antigen recognition profiles of the core candidate VHHs, homology models of H9-HA, N2-NA, and the five selected VHHs were generated using Swiss-Model and SAVE v6.0, followed by molecular docking and binding affinity predictions. Docking analyses revealed that HA-specific VHHs (L1-2, L1-4, A5) preferentially targeted the conserved stem/stalk region of H9-HA (Figure 3A–C). NA-specific VHHs (L1-3, L2-2) engaged regions near the enzymatic active site of N2-NA (Figure 3D,E) [42,43]. The binding interfaces were characterized by extensive hydrogen bonding, salt bridges, and non-bonded interactions, with buried surface areas ranging from 1020 to 1500 Å^2^, consistent with canonical nanobody–antigen interaction features (Table S2).

**Figure 4:**
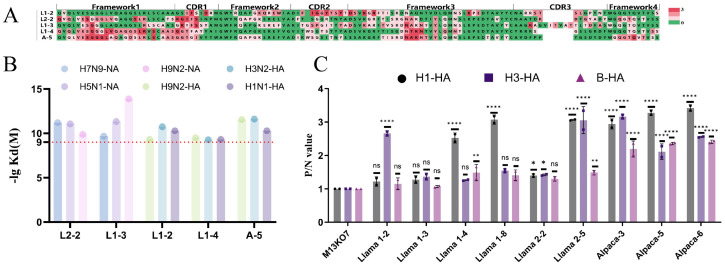
Cross-subtype binding predictions of selected VHHs. (**A**) Heatmap showing the number of antigen types potentially contacted by individual amino acids across VHH structural regions (FR1, CDR1, FR2, CDR2, FR3, CDR3, FR4). Color gradient represents contact frequency: red, high; green, low. (**B**) Bar and scatter plots depicting predicted binding affinities (−log Kd) and binding free energies (ΔG) of selected VHHs to HA and NA. The dashed red line at −log Kd = 9 indicates the minimum threshold within the expected range for nanobody–antigen interactions. Data are derived from docking predictions and should be interpreted qualitatively. (**C**) Cross-reactivity of selected clones against heterologous HA subtypes (H1, H3, influenza B). Several clones, including L1-2 and L1-4, display partial cross-subtype reactivity. Data represent mean ± SD of three independent measurements. Statistical significance: * *p* < 0.05, ** *p* < 0.01, **** *p* < 0.0001; “ns” indicates no significant difference.

Cross-subtype binding analysis indicated that the HA-specific VHHs could potentially recognize conserved epitopes across H1, H3, and *influenza B virus* HAs, while NA-specific VHHs showed potential cross-subtype recognition of N1 and N9 neuraminidases (Figures S4–S6). While the complementarity-determining regions (CDRs) served as the primary antigen-binding sites, several framework residues contributed to epitope recognition, acting as critical auxiliary determinants for cross-subtype binding (Figure 4A). Predicted dissociation constants (Kd), calculated via PRODIGY, fell within the expected range for nanobody–antigen interactions [44] (Figure 4B), suggesting robust antigen-binding potential. Importantly, ELISA experiments confirmed that HA-specific phage-displayed VHHs could cross-bind H1, H3, and influenza B HAs, validating the computational predictions (Figure 4C). These computational and experimental results together support the potential of HA-specific VHHs for cross-subtype reactivity.

### 3.4. Construction and Expression Characterization of Yeast-Secreted VHH-IgY Fc Fusion Proteins

**Figure 5:**
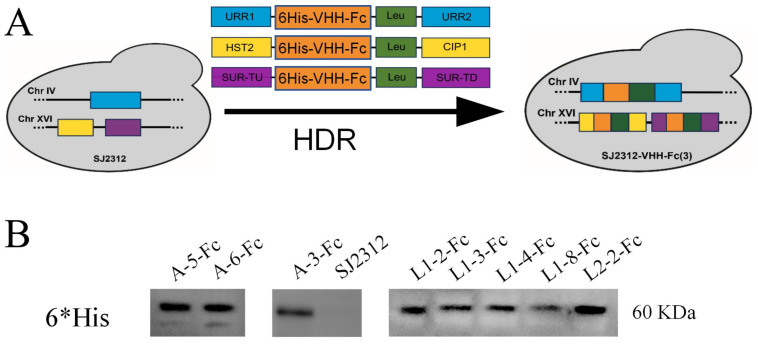
Expression and detection of yeast-secreted VHH-Fc fusion proteins. (**A**) Schematic of the homologous recombination strategy for stable integration of VHH-Fc constructs into the *S. cerevisiae* genome. Different colors represent distinct homologous recombination cassettes integrated at three independent genomic loci in *S. cerevisiae*: blue indicates the URR1/URR2-flanked cassette, yellow indicates the HST2/CIP1-flanked cassette, purple indicates the SUR-TU/SUR-TD-flanked cassette, orange indicates the 6His-VHH-Fc expression construct, and green indicates the *Trp* marker. (**B**) Western blot of culture supernatants from recombinant *S. cerevisiae* expressing VHH-Fc proteins. Bands at ~60 kDa correspond to the expected molecular weight of VHH-Fc fusions.

To enable efficient and scalable expression of the candidate VHHs for potential application in poultry influenza prevention, the five core VHHs were fused to the chicken IgY Fc domain. Recombinant expression vectors were constructed and genomically integrated into the cell wall-deficient *S. cerevisiae* strain SJC1 [45], establishing a yeast-based secretory expression platform (Figure 5A). Western blot analysis detected a specific protein band of approximately 60 kDa in the culture supernatant, consistent with the predicted molecular weight of the VHH-IgY Fc fusion, confirming successful extracellular secretion (Figure 5B). BCA protein quantification revealed secretion yields of 15–20 mg/L, comparable to typical yields of VHHs expressed in *E. coli* [46], demonstrating that the yeast secretion system provides efficient protein production with potential for scalable application.

Combined with the positive binding results from the previous phage ELISA, it was confirmed that the yeast-secreted VHH-IgY Fc fusion proteins retained the antigen-binding motifs of the VHHs, providing a reliable protein foundation for subsequent in vitro functional validation.

### 3.5. In Vitro Functional Validation of VHH-IgY Fc Fusion Proteins

**Figure 6:**
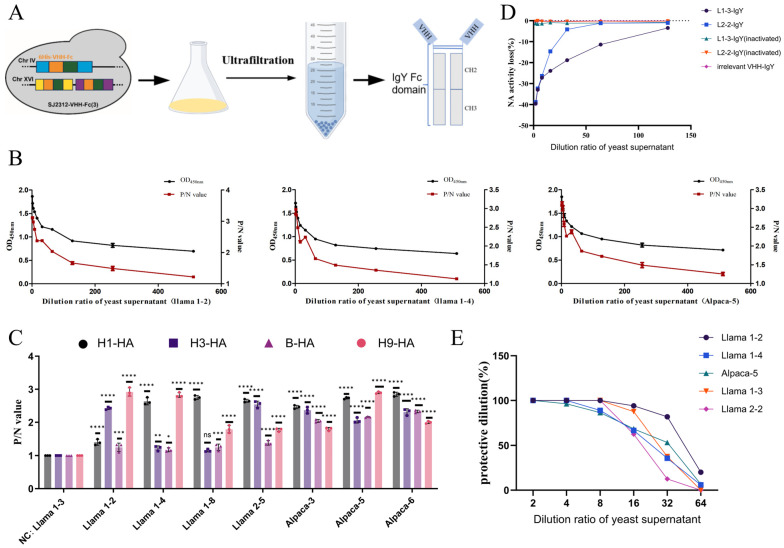
Functional validation of yeast-expressed VHH-Fc proteins. (**A**) Schematic representation of VHH-Fc fusion protein binding to influenza antigens. The color-coded curves correspond to different constructs as defined in the legend of Figure 5A. (**B**) ELISA binding curves of purified VHH-Fc proteins against H9-HA and heterologous HA subtypes (H1, H3). OD_450_ decreases with dilution, indicating high-affinity binding. (**C**) NA inhibition assay (MUNANA substrate) showing dose-dependent inhibition by L1-3 and L2-2 VHH-Fcs. IC_50_ values indicated. Irrelevant VHH-Fc used as negative control. Statistical significance: * *p* < 0.05, ** *p* < 0.01, *** *p* < 0.001, **** *p* < 0.0001; “ns” indicates no significant difference compared to the negative control (NC). (**D**) Cross-subtype binding of HA-targeting VHH-Fcs confirmed by ELISA, demonstrating recognition of conserved HA stem epitopes. The dotted dashed line represents 0% NA activity loss (100% residual activity), serving as a reference baseline to compare the relative enzymatic inhibition across different dilution ratios. Data represent mean ± SD of three independent experiments. (**E**) The protective activity of recombinant yeast supernatants was assessed using a two-fold serial dilution assay with eight replicate wells per dilution. The 50% protective dilution (PD_50_) was determined according to the Reed–Muench method. Briefly, cumulative counts of wells exhibiting cytopathic effect (CPE) or no CPE were tabulated across the dilution series, and the PD_50_ was interpolated based on the proportionate distance from the 50% protection threshold.

This study systematically evaluated the antigen-binding specificity, cross-subtype recognition, neuraminidase (NA) inhibition, and anti-H9N2 virus-neutralizing activity of the VHH-IgY Fc fusion proteins (Figure 6A) using ELISA, MUNANA fluorescence assays, and virus neutralization tests. These experiments also validated the structural predictions described in Section 3.3.

#### 3.5.1. Antigen Binding and Cross-Subtype Recognition

Indirect ELISA revealed that the HA-specific VHH-IgY Fc fusion proteins selectively bound H9-HA (Figure 6B) and exhibited robust cross-reactivity with H1 and H3 subtype HAs, as well as *influenza B virus* HA, in strong agreement with the predicted cross-subtype binding potential. In contrast, the NA-specific VHH-IgY Fc fusions displayed exclusive binding to N2-NA, confirming that the fusion proteins preserved both the antigen-binding specificity of the parental VHHs and their cross-subtype recognition capabilities (Figure 6C).

#### 3.5.2. Neuraminidase Activity Inhibition

NA activity assays demonstrated that the NA-specific VHH-IgY Fc fusions (L1-3, L2-2) inhibited N2-NA enzymatic activity in a dose-dependent manner, with IC_50_ values ranging from 0.69 to 1.72 μg/mL. An irrelevant VHH-IgY Fc control exhibited no significant inhibition, confirming that the observed NA inhibition was target-specific and functionally effective (Figure 6D).

#### 3.5.3. Anti-H9N2 Virus Neutralization

Virus neutralization assays showed that HA-specific clones L1-2, L1-4, and A5 achieved PD_50_ values of 1:45, 1:23, and 1:33, respectively, while NA-specific clones L1-3 and L2-2 had PD_50_ values of 1:27 and 1:18 (Figure 6E). All five candidate fusion proteins effectively prevented H9N2 virus infection of MDCK cells, with neutralizing activity positively correlating with the binding affinities observed in phage ELISA. These results demonstrate that VHH-IgY Fc fusions targeting the conserved HA stalk and NA active sites exhibit potent in vitro antiviral activity.

In summary, following fusion with chicken IgY Fc and secretion via the yeast expression system, the core VHHs retained their full antigen-binding capacity, enzymatic inhibition, and virus-neutralizing functions, while also demonstrating cross-subtype recognition, underscoring their potential as versatile antiviral agents for influenza control in poultry.

## 4. Discussion

Antigenic drift and antigenic shift of influenza viruses enable them to continuously evade host immune pressure, thereby limiting the efficacy of existing vaccines and antiviral drugs [47]. This biological characteristic necessitates that neutralizing agents against influenza viruses possess sufficient breadth to counteract threats from multiple subtypes. Nanobodies have garnered widespread attention in influenza research in recent years due to their small molecular weight, structural stability, and ability to recognize conformational epitopes [17]. For instance, a nanobody named E10, targeting the conserved head region of the H7 subtype hemagglutinin (HA), was identified from immunized alpacas and demonstrated heterosubtypic protection [24]. Another study reported nanobodies targeting the stem region of H1 subtype HA, among which E13 protected mice from H1N1 challenge at extremely low doses [48]. However, the identification of these nanobodies relied on immunization strategies or recombinant protein antigens, and their breadth was largely limited to certain subtypes of *influenza A virus*, with rare reports on protection against *influenza B virus* [18].

This study establishes a technology platform for the rapid generation of cross-subtype–binding anti-influenza nanobodies, eliminating the need for immunization. Unlike previous approaches using recombinant monomeric HA or neuraminidase (NA) proteins for screening, this study employed whole cells infected with H9N2 as the screening matrix. By exploiting the budding stage of the viral infection cycle, naturally conformed trimeric HA and tetrameric NA displayed on the cell membrane were captured [32,49]. This strategy effectively avoided issues such as false positives or reduced affinity caused by conformational changes or epitope loss in recombinant antigens, thereby improving the efficiency of isolating functional antibodies. Through multiple rounds of panning and removal of nonspecific adsorption, the obtained VHH clones exhibited differential binding signals in enzyme-linked immunosorbent assays (ELISA). Molecular docking further suggested that CDRs and FR residues collectively contributed to antigen recognition, with FR residues playing a significant role in binding to conserved regions.

By integrating experimental screening with structural prediction, this study identified VHH clones exhibiting both strong binding capacity and favorable interaction modes. Binding activity analysis revealed that HA-specific VHHs targeted conserved epitopes in the HA stem region, enabling broad recognition of HA antigens from H1 and H3 subtypes of *influenza A virus* as well as *influenza B virus*. In contrast, NA-specific VHHs targeted the enzyme active center, demonstrating potent neuraminidase inhibition (IC_50_ = 0.69–1.72 μg/mL) and virus neutralization capacity (PD_50_ = 1:18–1:45). This dual-target design strategy not only broadens the antiviral spectrum but also reduces the risk of viral resistance through single epitope mutations.

To address the limitation of rapid in vivo clearance and short half-life due to the small molecular weight (~15 kDa) of nanobodies [50], the selected VHHs were fused with the Fc fragment of avian IgY, a characteristic antibody type in birds. Existing literature indicates that Fc fusion can extend antibody half-life and enhance immune effector functions [27]. Furthermore, the compatibility of IgY Fc with the avian immune system offers unique advantages for developing avian-specific biologics and may support future exploration of mucosal immunization in poultry [21,22].

Theoretically, IgY Fc-mediated ADCC and/or ADCP could promote viral clearance in birds by recruiting avian phagocytes and natural killer-like cells [51]; however, the underlying molecular mechanisms in avian immune systems remain poorly understood—a key gap in the field. Future efforts will focus on establishing a standardized avian-specific ADCC/ADCP assay platform, elucidating whether IgY Fc fusion enhances VHH antiviral activity via effector functions, and to systematically evaluate these properties in waterfowl-derived optimized antibodies.

Camelid VHHs may elicit immune responses in chickens due to evolutionary divergence from avian immunoglobulins. Nevertheless, several factors mitigate this risk: (1) the small molecular weight of VHHs (~15 kDa); (2) fusion with chicken IgY Fc enhances immunological compatibility; and (3) high specificity and low off-target binding observed in vitro. Future in vivo studies in SPF chickens will systematically evaluate anti-VHH and anti-IgY Fc antibody generation following single or repeated dosing.

In vivo pharmacokinetic, tissue distribution, and safety evaluations have not yet been conducted but are underway. Similarly, in vivo protective efficacy via SPF chicken challenge—the gold standard for poultry biologics will be assessed in the future. The ongoing H9N2 SPF chicken challenge will assess survival, clinical scores, weight gain, viral load, viral shedding, and respiratory tract histopathology.

To meet the requirements for large-scale production and biosafety, this study employed food-grade *S. cerevisiae* to express VHH-IgY Fc chimeric antibodies. The yeast expression system combines the high yield of prokaryotic systems with the post-translational modification capabilities of eukaryotic systems, avoiding issues such as inclusion body formation and misfolding commonly observed in *E. coli* expression [52]. Secretory expression yields of 15–20 mg/L were achieved in this study, comparable to the approximately 15 mg/L yield of prokaryotic *E. coli* systems. However, antibodies produced in yeast retained complete binding activity and broad-spectrum neutralization capacity, with the added benefits of low culture costs, absence of endotoxin contamination, and scalability for production. Previous studies have confirmed the utility of yeast systems for rapid screening and expression of functional antibodies [53], and the successful commercialization of products such as the HPV vaccine underscores the safety and controllability of yeast as an expression host.

In summary, this study integrates cell-based matrix screening, dual-target design, IgY Fc fusion, and yeast expression to establish a non-immunization, rapidly responsive platform for developing broad-spectrum anti-influenza nanobodies. The resulting VHH-IgY Fc chimeric antibodies demonstrate advantages in binding breadth, neutralization activity, and production accessibility, positioning them as promising candidates for biologics aimed at controlling multi-subtype avian influenza viruses. Future studies should further validate their in vivo protective efficacy, conduct affinity maturation optimization, and explore the feasibility of large-scale fermentation processes. In addition, future work will focus on developing waterfowl IgG-type VHH fusion antibodies, while preserving antigen specificity and neutralization potency, to extend the potential of these candidates for waterfowl therapeutic applications. This platform strategy also provides a referenceable technical pathway for developing antibody drugs against other highly mutable viruses.

## 5. Conclusions

This study presents a generalizable, immunization-independent pipeline for rapid development of camelid-derived nanobodies (VHHs). By integrating cell-based screening, dual-target design, structural prediction, and yeast-based secretion, the platform enables efficient identification and production of functional VHH–Fc constructs. Selected VHH–Fc clones demonstrated potent dual-target antiviral activity, high-yield secretion, and cross-subtype binding, collectively confirming their functional efficacy. Notably, this is the first report of camelid VHHs fused to chicken IgY Fc for targeting influenza HA and NA. Overall, this approach provides a versatile and scalable cell-based platform with practical relevance for poultry antiviral interventions, bridging molecular engineering and translational application.

## Figures and Tables

**Table 1:** Primer sequences of nested PCR.

Primer Name	Sequence (5′→3′)
CALL-F	GTCCTGGCTGCTCTWCTACAAGGTGTC
CALL-R	GGTACGTGCTGTTGAACTGTTCC
VHH-SfiI-F	TCCTTTCTATGCGGCCCAGCAGGTGCAGCTGGTAGAGTC
VHH-NotI-R	CGGCACCGGCGCACCTGCTGAGGAGACGGTGACCTGGGT
CALL-F	GTCCTGGCTGCTCTWCTACAAGGTGTC
CALL-R	GGTACGTGCTGTTGAACTGTTCC

## Data Availability

The original contributions presented in this study are included in the article/Supplementary Materials. Further inquiries can be directed to the corresponding authors.

## Supplementary Materials

The following supporting information can be downloaded at: https://www.mdpi.com/article/10.3390/cells15080655/s1. Figure S1: Colony PCR Validation of VHH Insertions; Figure S2: Alignment of VHH FR2 and CDR regions in alpaca and llamas. Figure S3: Nine unique VHH sequence alignment of phage libraries; Figure S4: Molecular structures depicting interactions of variable domain heavy-chain fragments (VHHs) with hemagglutinin (HA) from different *influenza A virus* subtypes. Figure S5: Molecular structures depicting interactions of variable domain heavy-chain fragments (VHHs) with neuraminidase (NA) from different *influenza A virus* subtypes; Figure S6: Comprehensive Interaction Mapping: Long composite diagram showing residue-specific interactions of VHHs with HA/NA subtypes. Figure S7: Western blot of culture supernatants from recombinant *S. cerevisiae* expressing VHH-Fc proteins; Figure S8: Raw OD_450_ nm values for key ELISA experiments in the manuscript. Table S1: Titers of eluted phages from three rounds of cell-based panning; Table S2: Buried surface area and interaction parameters of VHH-HA/NA complexes predicted by molecular docking.

## Abbreviations

The following abbreviations are used in this manuscript:

VHH	Variable Domains of Heavy Chain
HA	Hemagglutinin
NA	Neuraminidase
IAV	*Influenza A virus*
MDCK	Madin-Darby Canine Kidney
PBMCs	Peripheral Blood Mononuclear Cells
CDR3	Complementarity Determining Region 3
OD_450_	Optical density at 450 nm
CFU	Colony-Forming Units
TCID_50_	50% Tissue Culture Infectious Dose
PD_50_	50% Protective Dose
IC_50_	50% Inhibitory Concentration
ADCC	Antibody-Dependent Cellular Cytotoxicity
CDC	Complement-Dependent Cytotoxicity
