# Supplementary material for: From Phage Display to Yeast Secretion: Developing Fc-Fused Nanobodies Against Influenza Virus
Source: Cells. 2026 Apr 8;15(8):655. doi: 10.3390/cells15080655 (PMC13114647; doi:10.3390/cells15080655)
Supplement: Supplementary file 1 [file cells-15-00655-s001.zip › cells-4226105-supplementary.pdf]

# *Supplementary Material*

## 1 Supplementary Figures

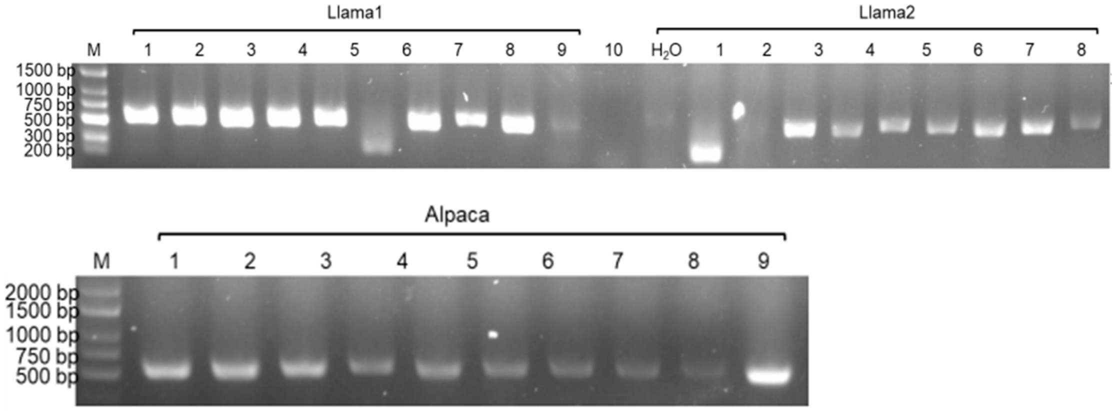


Figure S1. Colony PCR Validation of VHH Insertions: Successful construction of the library was confirmed in both species, with 90% of the positive clones in llamas and 100% in alpacas.

**A.**

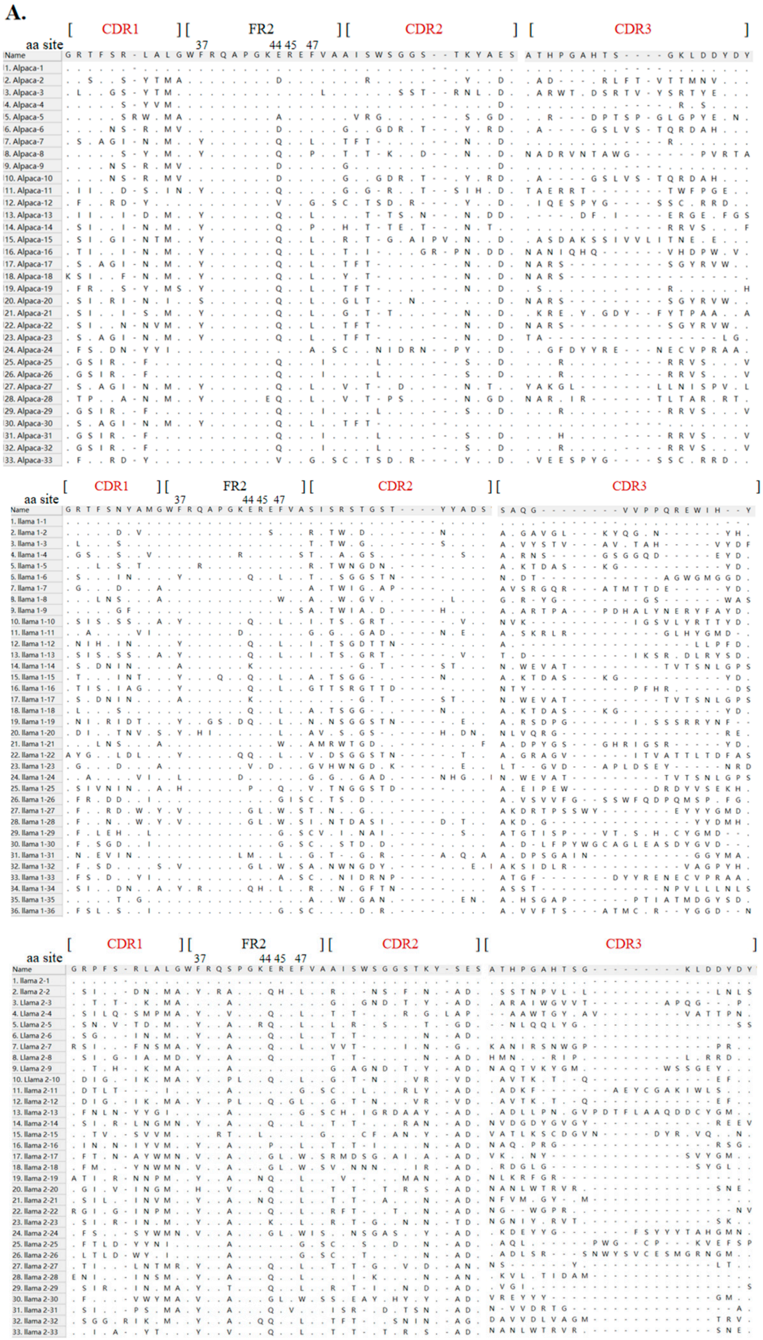

B.

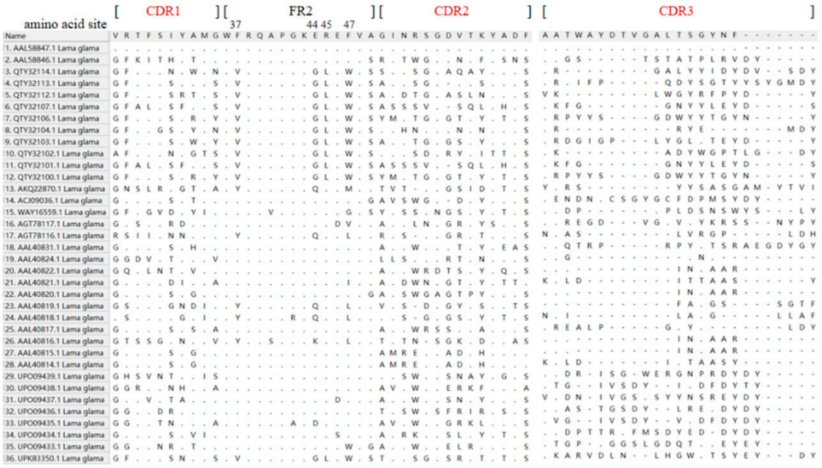


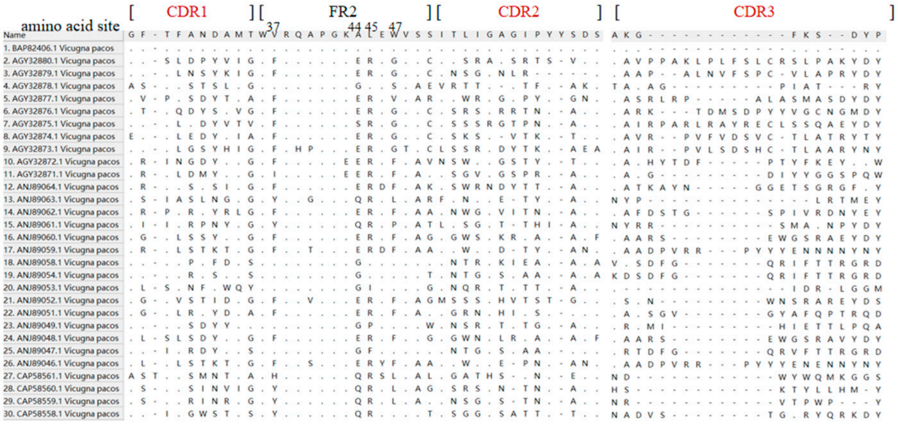


C.

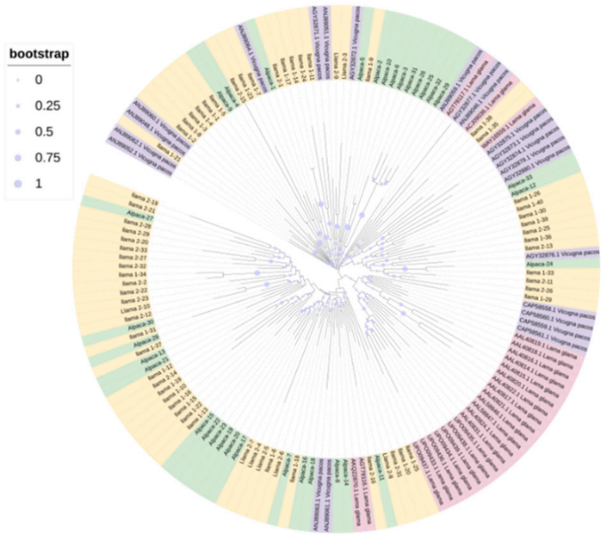


Figure S2. Alignment of VHH FR2 and CDR regions in alpaca and llamas. A. Sequenced alpaca and camelid VHH. B. GenBank searches for alpaca and camelid VHH. C. Phylogenetic tree of amino acid sequences in FR regions of alpaca and llamas.

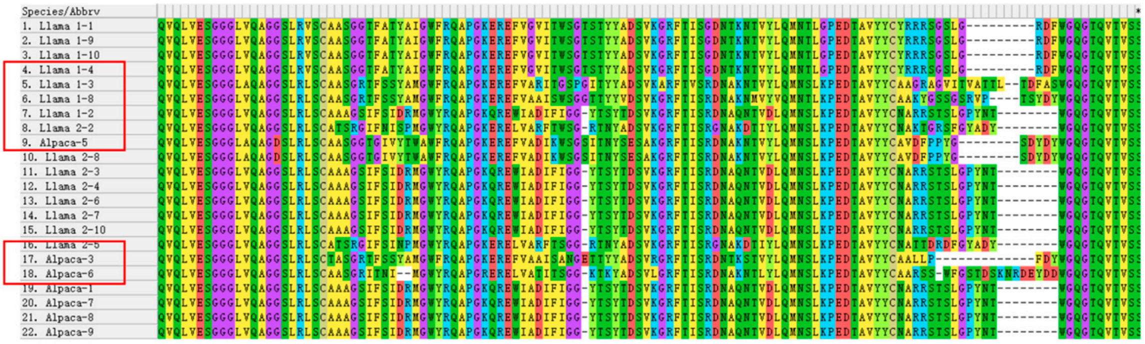


Figure S3. Nine unique VHH sequence alignment of phage libraries

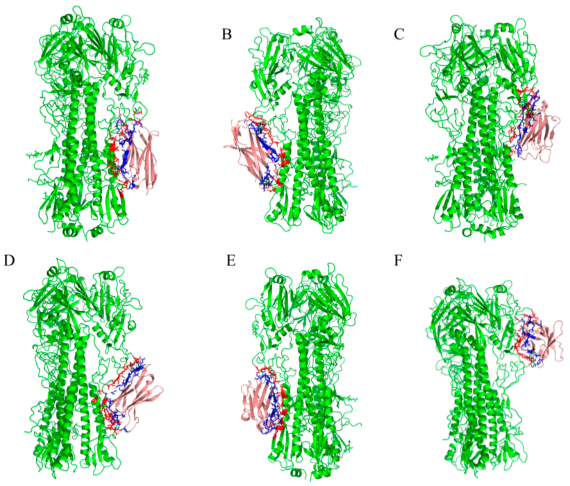


Figure S4. Molecular structures depicting interactions of variable domain heavy chain fragments (VHHs) with hemagglutinin (HA) from different influenza A virus subtypes. (A) Structural model of H3N2 - HA (H3) with VHH L1 - 2; (B) Structural model of H1N1 - HA (H1) with VHH L1 - 2; (C) Structural model of H3N2 - HA (H3) with VHH L1 - 4; (D) Structural model of H1N1 - HA (H1) with VHH L1 - 4; (E) Structural model of H3N2 - HA (H3) with VHH A - 5; (F) Structural model of H1N1 - HA (H1) with VHH A - 5.

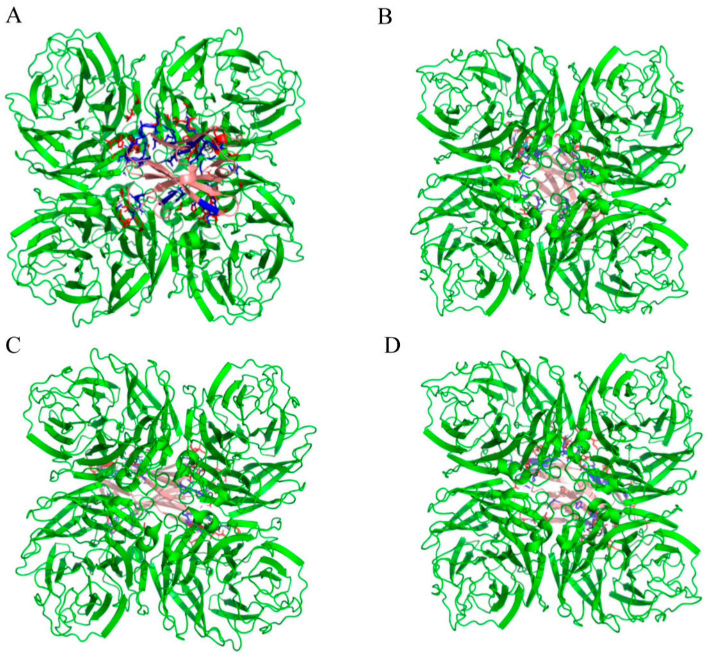


Figure S5. Molecular structures depicting interactions of variable domain heavy chain fragments (VHHs) with neuraminidase (NA) from different influenza A virus subtypes.(A) Docking model of H1N1 - NA (N1) with VHH L1 - 3; (B) Docking model of H9N2 - NA (N9) with VHH L1 - 3; (C) Docking model of H1N1 - NA (N1) with VHH L2 - 2;(D) Docking model of H9N2 - NA (N9) with VHH L2 - 2.

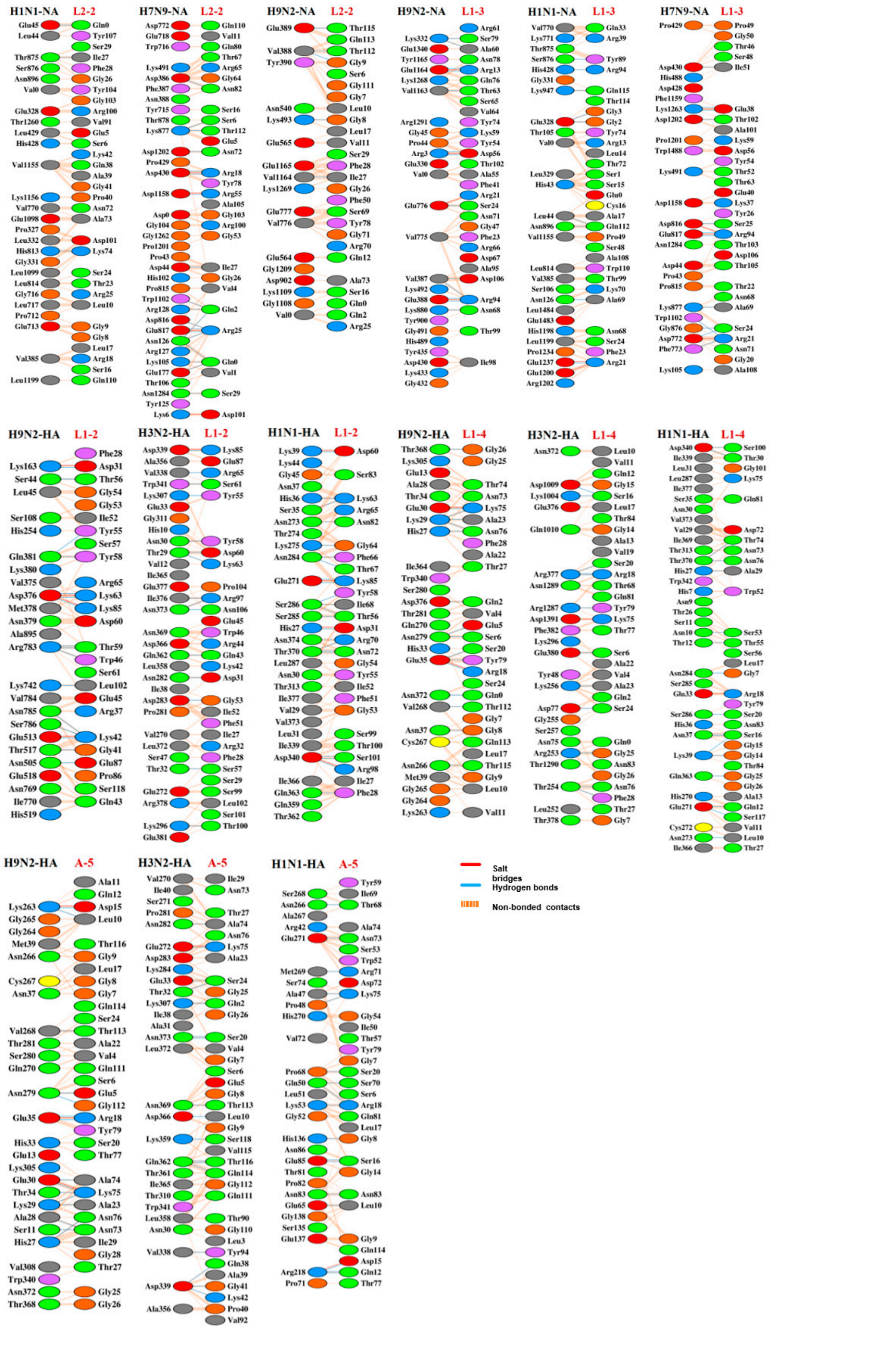


Figure S6. Comprehensive Interaction Mapping: Long composite diagram showing residue-specific interactions of VHHs (L2-2, L1-3, L1-2, L1-4, A-5) with H9N2/N1/H1 HA/NA subtypes. Colored circles indicate the interaction types (red: salt bridges; blue: hydrogen bonds; gray: non-bonded contacts).

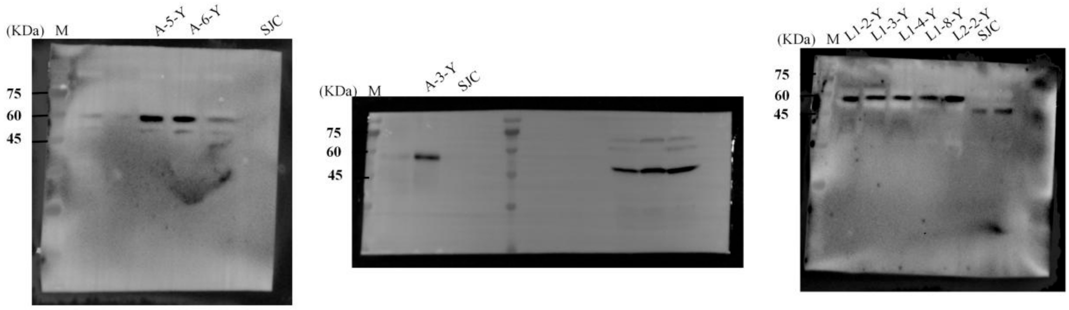


Figure S7. Western blot of culture supernatants from recombinant *S. cerevisiae* expressing VHH-Fc proteins. Bands at ~60 kDa correspond to expected molecular weight of VHH-Fc fusions.

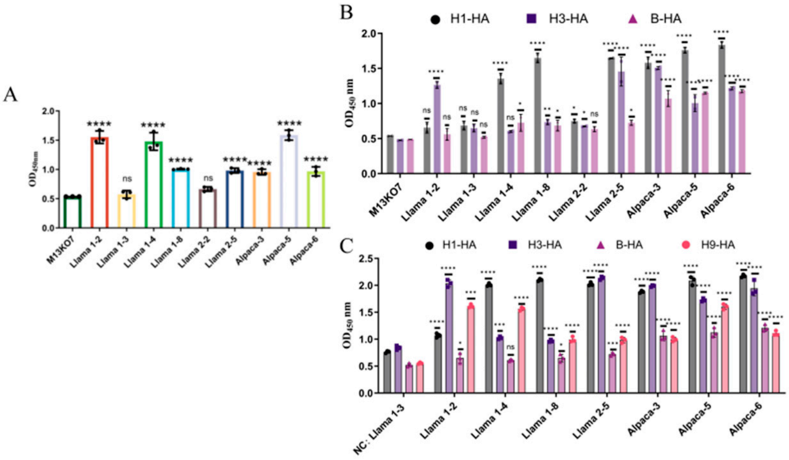


Figure S8: Raw $OD_{450}$ nm values for key ELISA experiments in the manuscript. (A) Raw $OD_{450}$ nm values corresponding to Figure 2C, showing the binding activity of 9 H9N2-specific VHHs to H9-HA antigen. (B) Raw $OD_{450}$ nm values corresponding to Figure 4C, demonstrating the cross-subtype binding activity of candidate VHHs to H1-HA, H3-HA, and B-HA antigens. (C) Raw $OD_{450}$ nm values corresponding to Figure 6C, verifying the binding specificity of VHH-IgY Fc fusion proteins to H9-HA, H1-HA, H3-HA, and B-HA antigens. All data are presented as mean ± SD from three independent biological replicates. For panels A and B, statistical significance was calculated relative to the M13KO7 helper phage negative control. For panel C, statistical significance was calculated relative to an irrelevant VHH-IgY Fc (Llama 1-3) used as the negative control (NC). Significance levels are indicated as follows: *P < 0.05, ***P < 0.001, ****P < 0.0001; ns, not significant.

## 2 Supplementary Tables

Table S1 Titers of eluted phages from three rounds of cell-based panning

| Library Name | Incubated Virus Titer | Selection Round （PFU） | Incubated Virus Titer （PFU） |
|---|---|---|---|
| Llama-1-A | 1 | $1\times10^{12}$ | $1\times10^{5}$ |
| | 2 | $1\times10^{12}$ | $2.4\times10^{6}$ |
| | 3 | $1\times10^{12}$ | $2\times10^{7}$ |
| Llama-2-A | 1 | $1\times10^{12}$ | $5.52\times10^{5}$ |
| | 2 | $1\times10^{12}$ | $2.02\times10^{6}$ |
| | 3 | $1\times10^{12}$ | $9.9\times10^{6}$ |
| Alpaca-A | 1 | $1\times10^{12}$ | $2.7\times10^{5}$ |
| | 2 | $1\times10^{12}$ | $7.05\times10^{6}$ |
| | 3 | $1\times10^{12}$ | $3.5\times10^{7}$ |
| M13KO7 | 1 | $1\times10^{12}$ | $1.75\times10^{2}$ |
| | 2 | $1\times10^{12}$ | $5\times10^{2}$ |
| | 3 | $1\times10^{12}$ | $1.4\times10^{2}$ |

Three nanobody phage display libraries (Llama-1, Llama-2 and Alpaca) were diluted with PBS to $1\times10^{12}$ PFU/mL. 1 mL of phage dilution was added per well to MDCK cells infected with H9N2 for 48 h. After incubation for 45 min, specific phages were eluted by freeze－thaw cycles. M13KO7 at the same titer was used.

Table S2 Buried surface area and interaction parameters of VHH-HA/NA complexes predicted by molecular docking

| Chain | No. of interface residues | Interface area (Å2) | No. of salt bridges | No. of hydrogen bonds | No. of non-bonded contacts |
|---|---|---|---|---|---|
| H9N2-H9 | 24 | 1313 | 4 | 16 | 174 |
| L1-2 | 25 | 1396 | | | |
| H9N2-H9 | 26 | 1299 | 2 | 16 | 184 |
| L1-4 | 28 | 1227 | | | |
| H9N2-H9 | 26 | 1388 | 3 | 15 | 180 |
| A-5 | 32 | 1349 | | | |
| H9N2-NA | 17 | 1158 | - | 5 | 146 |
| L2-2 | 26 | 1021 | | | |
| H9N2-NA | 25 | 1506 | 4 | 13 | 190 |
| L1-3 | 29 | 1444 | | | |
